# Management of older adults with hip fractures in India: a mixed methods study of current practice, barriers and facilitators, with recommendations to improve care pathways

**DOI:** 10.1007/s11657-017-0344-1

**Published:** 2017-06-02

**Authors:** Santosh Rath, Lalit Yadav, Abha Tewari, Tracey Chantler, Mark Woodward, Prakash Kotwal, Anil Jain, Aparajit Dey, Bhavuk Garg, Rajesh Malhotra, Ashish Goel, Kamran Farooque, Vijay Sharma, Premila Webster, Robyn Norton

**Affiliations:** 1 The George Institute for Global Health, University of Oxford, Oxford, UK; 2 IGHI, Imperial College, Kensington, London, SW7 2AZ UK; 3 The George Institute for Global Health, New Delhi, India; 4 London School of Hygiene & Tropical Medicine, London, UK; 5 The George Institute for Global Health, University of Sydney, Sydney, Australia; 6 Department of Orthopaedics, All India Institute of Medical Sciences (AIIMS), New Delhi, India; 7 Department of Orthopaedics, University College of Medical Sciences, Delhi, India; 8 Department of Geriatrics, All India Institute of Medical Sciences, New Delhi, India; 9 Department of Medicine, University College of Medical Sciences, Delhi, India; 10 JPN Trauma Centre, All India Institute of Medical Sciences, New Delhi, India; 11 School of Public Health, Nuffield Department of Population Sciences, University of Oxford, Oxford, UK

**Keywords:** Hip fracture, India, Care pathways, Fragility fracture, Mixed methods

## Abstract

**Summary:**

Evidence-based management can reduce deaths and suffering of older adults with hip fractures. This study investigates the evidence-practice gaps in hip fracture care in three major hospitals in Delhi, potential barriers and facilitators to improving care, and consequently, identifies contextually appropriate interventions for implementing best practice for management of older adults with hip fractures in India.

**Purpose:**

Hip fracture in older adults is a significant public health issue in India. The current study sought to document current practices, identify barriers and facilitators to adopting best practice guidelines and recommend improvements in the management of older adults with hip fractures in Delhi, India.

**Methods:**

This mixed methods observational study collected data from healthcare providers, patients, carers and medical records from three major public tertiary care hospitals in Delhi, India. All patients aged ≥50 years with an X-ray confirmed hip fracture that were admitted to these hospitals over a 10-week period were recruited. Patients’ data were collected at admission, discharge and 30 days post-injury. Eleven key informant interviews and four focus group discussions were conducted with healthcare providers. Descriptive data for key quantitative variables were computed. The qualitative data were analysed and interpreted using a behaviour change wheel framework.

**Results:**

A total of 136 patients, 74 (54%) men and 62 women, with hip fracture were identified in the three participating hospitals during the recruitment period and only 85 (63%) were admitted for treatment with a mean age of 66.5 years (SD 11.9). Of these, 30% received surgery within 48 h of hospital admission, 95% received surgery within 39 days of hospital admission and two (3%) had died by 30 days of injury. According to the healthcare providers, inadequate resources and overcrowding prevent adequate caring of the hip fracture patients. They unanimously felt the need for protocol-based management of hip fracture in India.

**Conclusion:**

The development and implementation of national guidelines and standardized protocols of care for older people with hip fractures in India has the potential to improve both care and patient-related outcomes.

**Electronic supplementary material:**

The online version of this article (doi:10.1007/s11657-017-0344-1) contains supplementary material, which is available to authorized users.

## Introduction

Hip fractures in older adults have significant implications for morbidity, mortality, hospital utilization and the cost of care in the community [1]. The annual healthcare bill is around 12 billion USD for the management of 250,000 individuals with hip fractures in the USA [2, 3] and around 3 billion USD for the care of 70,000 older patients with hip fractures in the UK [4, 5]. A report on India in 2004 estimated an annual incidence of 600,000 osteoporotic hip fractures [6], and this was expected to increase significantly by 2026, as the share of people over 60 years rises to 12.4% of 1.36 billion population [7–9]. Adoption of protocol-based care and clinician-led quality improvement initiatives where audit plays an important role have demonstrated a significant reduction in the 30-day and 1-year mortality rates following hip fracture injury among adults aged 60 years [4, 5, 10–12]. As a consequence, these audits have triggered worldwide interest in protocol-based multidisciplinary care for the management of older adults with hip fracture. Similar audits have recently begun in Ireland, Australia and New Zealand, Hong Kong and Canada [13–15].

The global burden of hip fractures is likely to increase significantly from an estimated 1.7 million in 1990 to 6.3 million in 2050 [16, 17]. These increases are primarily the consequence of improved life expectancy, especially in emerging economies, and it is projected that by 2050 nearly half of all hip fractures will occur in Asia, particularly in India and China [16, 18, 19]. Due to limited healthcare resources, nearly 2 billion people worldwide lack access to surgical care [20, 21]. High-income countries (HICs) have mean of 14 operating rooms and 45 trained surgeons per 100,000 population. In contrast, low- and middle-income countries (LMICs) have less than two operating rooms and less than one trained surgeon per 100,000 population [22]. Following a hip fracture, use of health services extends beyond the initial hospitalization for at least 1 year, with follow-up care accounting for the majority of health costs [21–24]. Patients, therefore, must be managed effectively and efficiently according to resource availability [25]. India does not have a universal healthcare system for all its citizens. Most healthcare expenses are paid out of pocket by patients and their families, rather than through insurance. According to the National Family Health Survey-3, the private medical sector remains the primary source of healthcare for 70% of households in urban areas and 63% of households in rural areas. But for poor and vulnerable people, the public sector remains the healthcare system to access as they cannot afford private medical care. Moreover, there is no public pre-hospital care for trauma in India. Rashtriya Swasthaya Bima Yojana (RSBY), a health insurance scheme, initially launched for below poverty line (BPL) households, now covers other defined categories of unorganized workers. But this community insurance scheme covers only a proportion of the population and defined diseases requiring hospitalization. Besides, the implementation of this scheme is varied in nature across different states in India [26, 27]. Early adoption of best practice guidelines and protocol-based care in low- and middle-income countries may have the potential to reduce the risk of mortality and cost of care and improve quality of life for older adults with hip fracture.

Our long-term aim is to facilitate the implementation of best practice for the management of older adults with hip fractures in India, to moderate the impact of this injury in the coming decades. In India, a systematic approach is required to set minimum standards and adoption of protocol-based care pathways for the management of older people with hip fractures [28]. In the first instance, this requires comparison of current practice with recognized best practice standard to help identify practice gaps [23]. It also requires the identification of barriers and facilitators to adopting best practice to enable the development of strategies [24] to implement evidence-informed protocols for care [25].

The study aims to document current practices, barriers and facilitators to adopting best practice guidelines and consequently makes recommendations for improving the management of older adults with hip fractures in Delhi, India.

## Methods

### Study design and setting

This mixed methods observational study collected quantitative and qualitative data concurrently [29] from healthcare providers (HCPs), patients, carers and medical records from three major public tertiary care hospitals in Delhi, India, from September 2014 to March 2015.

The study sites were selected purposively. Delhi is the second most populous city in India with a population of nearly 17 million and provides healthcare services, both for its population, from surrounding states and country-wide referrals. Surgical services at the district are often limited to caesarean sections and abdominal surgery, with limited orthopaedic service capacity, mainly for managing simple road traffic injuries. Geriatric patients with hip fractures and complex orthopaedic injuries are therefore usually self-referred to tertiary care centres in Delhi from the surrounding states. The National Institutes receive patient from all over the country. There are over 10 public tertiary trauma care hospitals in Delhi, some funded by the Government of India and the others by the local Delhi government. The private trauma care hospitals far outnumber the public hospitals and provide substantial trauma care in the Delhi region. Estimates are that over 70% of surgical care in India are provided by private care.

Prior to the selection of hospital sites, a stakeholder event was organized for representatives of major tertiary care hospitals in Delhi. The aims and objectives of the proposed study were discussed during this event and the sites, which subsequently agreed to participate, were selected for the study. The study sites included three Government tertiary healthcare centres. Two of these, the All India Institute of Medical Sciences (AIIMS) and Jai Prakash Narayan Apex Trauma Centre (JPNATC), are national referral tertiary care centre funded by the Ministry of Health and Family Welfare, Government of India. The third, Guru Teg Bahadur Hospital, the associated teaching hospital of the University College of Medical Sciences (UCMS), University of Delhi, is funded by local government and receives patients from north Delhi and surrounding areas.

### Data collection and management

All hospitalized patients aged ≥50 years with an X-ray confirmed hip fracture [30] were approached to participate in the study over a period of 10 weeks. Patients presenting with a hip fracture but who were not admitted were not recorded, so the numbers of hip fractures presenting across the three hospitals studied could not be determined. A designated resident in the department of orthopaedics at each study hospital was responsible for seeking informed consent from admitted patients.

Patients’ data were collected at two time points during the hospitalization period. Information on socio-demographic characteristics (age, sex, education, residence and occupation), cause and type of fracture, known pre-existing medical conditions, pre-fracture mobility and American Society of Anaesthesiologists (ASA) grade were collected on admission to hospital. The ASA grades are a widely used grading system for pre-operative health of surgical patients [31]. Information on in-hospital care pathway, orthopaedics and geriatrician co-management, time from admission to surgery, surgical procedure, complications including pressure ulcers, medication for bone health, falls prevention advice, in-hospital mortality, length of stay (LoS) and discharge destination were collected at the time of discharge from case logs (Appendices 1A and 1B). LoS is defined as the time from hospital admission to discharge. A 30-day post-injury follow-up was conducted through a telephone interview (Appendix 2). Of the three participating hospitals, one had an electronic hospital record system. In this hospital, hip fracture patients usually report to the emergency department, and depending upon the availability of beds, only a proportion of patients are admitted and others are referred to nearby hospitals.

For the qualitative data collection, the study participants were HCPs, including clinical leads, residents and nursing staff from the departments of orthopaedics, anaesthesia, geriatrics, medicine and physiotherapy involved in pre-operative, operative and post-operative care. Eleven key informant interviews (KIIs) and four focus group discussions (FGDs) with HCPs were conducted using interview schedule and FGD guide. These comprised of open-ended questions to obtain information on existing care pathways within their hospital setting and potential barriers and facilitators to adopting best practices (Appendices 3 and 4).

Two research staff, trained in qualitative research, conducted these KIIs and FGDs. Each FGD comprised of 8–10 participants. All the interviews and FGDs were conducted in English or Hindi (local language), or both, as appropriate. All the conversation/discussions were audio-recorded, transcribed and translated into English. The duration of the interviews and focus groups was 30–45 and 45–60 min, respectively.

The data were maintained and accessible by research staff at the George Institute for Global Health, India. Each site maintained a master sheet with identifying information during the enrolment period to ensure that multiple entries into the database were not made for the same patient. The KIIs and FGDs were recorded, transcribed verbatim and translated in English (where necessary), and the files were only accessible to the research team.

### Data analysis

#### Quantitative

The quantitative data were de-identified and entered into an Excel spreadsheet. The electronic data were kept password protected and stored on secure servers.

Frequency distributions were computed for key quantitative variables including demographic characteristics, type of fracture, treatment modality and type of anaesthesia. We calculated the time between injury and admission to hospital from data collected as ‘the day of injury’ and ‘day of admission’ [32]. The time interval from admission to surgery, length of stay and death at 30 days following the injury were determined.

#### Qualitative

The data were analysed using a thematic approach [33] and the files were uploaded to *NVivo* 9.2, a qualitative software programme which supports in organizing, indexing and coding of data.

**Fig. 1:**
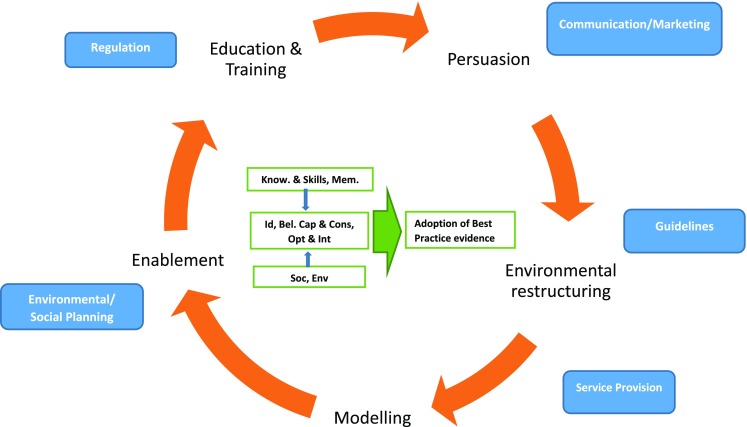
Behaviour change wheel (BCW)—adopting best practice evidence in the management of older adults with hip fracture in India. *Know.* knowledge; *Mem.* memory, attention and decision processes [capability]; *Id* social/professional role and identity; *Bel. Cap. & Cons* beliefs about capabilities and consequences; *Opt & Int* optimism and intentions [motivation]; *Soc* social influences; *Env* environmental context and resources [opportunity]. Sources of Behaviour *box and arrow in green*; Intervention Functions *Orange*; Policy Categories *Blue*

The identified themes were further interpreted using the behaviour change wheel framework (BCW) (Fig. 1). This allowed us to understand barriers and facilitators to adopting best practice guidelines through 10 theoretical domains, and to map contextually appropriate interventions. BCW is not a linear model and components within the behaviour system interact with each other to generate desired behaviour that in turn influences these components [34].

### Ethical considerations

Ethical approvals were obtained from the Institutional Ethics Committee (IEC) of all participating study sites (vide letter; IEC/NP-40/13.03.2014, RP-32/2014) and (vide letter 18.07.2014) and Health Ministry Screening Committee (HMSC) at Indian Council of Medical Research (vide letter No. 54/1/Indo- foreign/GER/2014-NCD-II, dated 10.10.2014). Written informed consent was obtained from study participants.

## Results

### Quantitative

A total of 136 patients, 74 (54%) men and 62 women, with hip fracture were identified in the three participating hospitals during the recruitment period. As per our inclusion criteria, all admitted patients were recruited to the study. Of the 136 patients, 51 were not admitted due to lack of beds (Table 3). Of the 85 admitted patients (63% of the total), 46 (54%) were men and 39 women, with a mean age of 66.5 years (SD 11.9), and all consented to participate in the quantitative part of the study. A fall from a standing height was the cause of the fracture in all admitted patients, and fractures were sustained on both sides of the body equally. Of those admitted to the orthopaedic ward, nearly half (48%) of the patients were admitted within 24 h of the injury and a fifth were admitted after 48 h.

The majority (65%) of fractures were inter-trochanteric, 29% were intra-capsular and 6% were sub-trochanteric fracture. All the older adults were independently mobile and only 13% of required mobility aids pre-fracture. The most common co-morbid conditions were hypertension (29% of patients) and type 2 diabetes mellitus in 12%. The ASA grades were documented in 50 patients with data missing for 40% of admitted hip fractures. Twenty-four (48%) patients had grade I, 22 (44%) had grade II, 2 (4%) had grade III and 3 (6%) had grade IV level of ASA. Only six patients (7%) were on bone protection medication which included bisphosphonates along with calcium and vitamin D supplements, prior to the fracture.

A total of 82 patients were operated for their hip fracture and almost all (98%) operated patients received a regional, spinal or epidural anaesthesia. Only 30% received surgery within 48 h of hospital admission, 27% were operated upon between 3 and 7 days, 22% between 8 and 14 days and 21% were operated upon 2 weeks after hospital admission. Intramedullary implants were used in 58% of inter-trochanteric fracture fixation and dynamic hip screws were used for the rest. The majority of patients with intra-capsular fractures received a cemented hemi-arthroplasty and only one received a total hip replacement.

At the study hospitals in Delhi, co-management of hip fractures by orthopaedic surgeons and geriatricians, osteoporosis assessment and medication, and falls assessment at discharge are not routinely practiced. Only nine patients (10%) received falls assessment and were prescribed anti-resorptive therapy along with calcium and vitamin D supplement at discharge. Data on pressure ulcers were not documented.

The mean LoS in hospital was 16 days (SD 10.91). All patients were discharged to their respective homes, as inpatient rehabilitation facilities were unavailable in these hospitals. Eleven patients (13%) were lost to follow-up at 30 days, some or all may have died. Two patients died by 30-day follow-up, one post-surgery in-hospital and the other following discharge from the hospital (Table 1).

**Table 1:** Demographics, fracture type, in-hospital care and 30-day mortality

Variable	Value	*n* (%)
Age (*n* = 85)	50–59	24 (28)
	60–69	21 (25)
	70–79	25 (29)
	80+	15 (18)
Gender (*n* = 85)	Male	46 (54)
	Female	39 (46)
Education (*n* = 77)	Illiterate	23 (30)
	Primary	25 (32)
	Secondary	16 (21)
	Graduate and above	13 (17)
Occupation (*n* = 79)	Employed	19 (24)
	Unemployed	13 (16)
	Retired	23 (29)
	Household work	22 (28)
	Others	2 (3)
Pre-fracture mobility (*n* = 82)	Without aid	71 (87)
	With one aid	11 (13)
Type of fracture (*n* = 85)	Intra-capsular	25 (29)
	Inter-trochanteric	55 (65)
	Sub-trochanteric	5 (6)
Anaesthesia (*n* = 82)	General	2 (2)
	Regional	80 (98)
Surgical procedure (*n* = 77)	Intramedullary nail	32 (42)
	Dynamic hip screw	23 (30)
	Cannulated cancellous screws	6 (8)
	Hemi-arthroplasty	15 (19)
	Total hip replacement	1 (1)
Time from injury to hospital admission (*n* = 85)	Less than 24 h	41 (48)
	24–48 h	29 (34)
	3–7 days	10 (12)
	>7 days	5 (6)
Time from hospital admission to surgery (*n* = 81)	Within 48 h	24 (30)
	3–7 days	22 (27)
	8–14 days	18 (22)
	More than 2 weeks	17 (21)
Length of stay (LoS) (*N* = 81)	2–3 days	2 (3)
	4–7 days	14 (17)
	8–14 days	30 (37)
	3–4 weeks	26 (32)
	>4 weeks	9 (11)
30-day post-injury mortality (*n* = 74)	Dead	2 (3)
	Alive	72 (97)

### Qualitative

#### Healthcare providers’ experience of hip fracture patients’ care pathways

According to HCPs, patients mainly originated from the national capital region of Delhi, which includes adjoining states, and were sometimes referred from hospitals lacking surgical facilities. Patients were taken to the hospital mainly by their relatives and at times by the patrolling police. Interviewees also reported that the majority of the patients seeking care in public hospitals are from low socio-economic status and have poor family support.

“Patients admitted to public hospitals were from quite different demographic and economic group (low socioeconomic) and the number of males are more than number of females”*—*orthopaedic surgeon (KII-8).

Most patients with acute fractures including hip fractures present at hospital either on the same day of injury or 2–3 days after the injury. Hip fracture patients generally present at the emergency department, where they are examined by a resident, admitted to the orthopaedic unit if beds are available, or referred to other hospitals. Many interviewees highlight that after admission, older people with hip fracture were frequently diagnosed for the first time with underlying co-morbidities such as diabetes, hypertension and renal and cardiac conditions.

“There maybe two factors… the family may be of the perception that it is a lost game and there is no point wasting money and let the person be at home. Another may be that our health system probably chooses the younger patient who has a better chance of recovery than in comparison to the older person”—geriatrics physician (KII-IX).

Almost all interviewees reported that in addition to orthopaedics, many other departments (e.g. medicine or geriatrics, anaesthesia, physiotherapy, endocrinology) were involved in treating hip fractures. After the diagnosis of hip fracture, the usual protocol of care is to assess the status of co-morbid conditions and try to optimize them for surgery, which involves a pre-anaesthesia check by a qualified anaesthetist. The majority of interviewees said that calcium and vitamin D supplements are part of standard care for all hip fracture patients. Anti-resorptive drugs are rarely prescribed and only to patients at risk for future osteoporotic fractures of the spine.

A geriatric consultant (KII1) opined: “One might consider prescribing zoledronic acid (anti-resorptive drug) if there is a suspicion of frequent falls or vertebral fractures”.

Interviewees drew attention to the lack of a ‘falls clinic’ at all three participating hospitals; however, according to a senior physiotherapist, assessment for falls prevention is provided if patients are referred for such advice or at times during follow-up. Patients are followed up 2 weeks post-surgery by an orthopaedic surgeon for suture removal and 3 months post-surgery for bone union and full weight bearing walking ability.

“Yes, we give falls prevention advise, we use some charts and boards and train them on walker … so that they don’t fall” (KII-5, senior physiotherapist).

#### Healthcare providers’ perspectives on barriers and facilitators to adopting best practice guidelines

The BCW framework (Fig. 1) was used to categorize the cited barriers and facilitators, using the constructs of capability (knowledge and skills; and memory, attention and decision processes), opportunity (social influences; environmental context and resources) and motivation (beliefs about capability and consequences; social and professional role; optimism and intentions).

##### Capability

Knowledge and skills—The majority of the HCPs believe that the management of hip fracture in older adults requires multidisciplinary care but lack consensus on early operative intervention and priority for surgery. Most orthopaedic surgeons were familiar with the existing international best practice guidelines for the management of older adults with hip fractures but some HCPs were not. Orthopaedic surgeons were viewed to be technically skilled in surgical treatment but less able to coordinate multidisciplinary care of older adults with hip fractures. Even though a geriatric department existed in one of the three hospitals, hip fracture patients with co-morbidities were rarely referred to this department. In effect, provision of specialist orthogeriatric care was non-existent across all three hospitals.

A senior orthopaedic consultant (KII3) commented: “It’s probably a failure on the part of the orthopaedic surgeon and geriatrician to appreciate the value of the multidisciplinary care and the lack of knowledge of integrated care pathways. Often the surgeon is keen on his operating procedures and the physician feels bothered being called in for a patient with a surgical problem and not a medical problem. There is some sort of failure to understand the necessity for collaborative work”.

“The geriatrician is never involved in the picture, mostly it’s the general physician who takes care of the patient’s comorbid conditions”—anaesthetist (KII6).

Memory, attention and decision processes—Almost all the HCPs felt that Delhi hospitals including their orthopaedic department were overcrowded due to a lack of definitive treatment for hip fractures at district hospitals. These HCPs further added that excessive workload from road traffic crashes deters priority for older adults with hip fractures. They also shared that lack of adequate number of beds leads to multiple hospital referrals and a bias against admitting sick patients who require prolonged length of stay. HCPs opined management protocols and priorities for care of older adults with hip fractures may differ between surgeons and the orthopaedic units in each hospital. They further recommended that adherence to standard treatment guidelines will reduce variations in decision-making and quality of care.

“Hospitals at all levels like a district hospital should be fully functional and not like the existing way. Even patients from smaller hospitals equipped with desired surgical facility are referring to our hospital (All agree). There are more patients but the doctors and OT (operation theatre) time is less”—HCPs views (FGD-3).

##### Opportunity

Social influences—According to the HCPs, the majority of the patients are from low socio-economic background and depend upon their family members to reach hospital. In addition, interviewees highlighted that the patients and their family members lack general health awareness and large numbers of patients with pre-existing co-morbid conditions are diagnosed for the first time while admitted for hip fractures. There is scant knowledge in the patient population, particularly from the adjoining states of Delhi about local health facilities, information on services and lack of triage to access appropriate care, leading to multiple hospital transfers. Most of the patients and relatives had no knowledge on the consequences of hip fracture injury in older people.

Environmental context and resources—According to the HCPs, inadequate staff, insufficient beds and overcrowding affect caring to the needs of the patients. The operating theatres (OTs) are inadequate to cater to the surgical workload. There are no dedicated trauma OTs or priority trauma lists. Compound fracture management and polytrauma patients consume most OT time. Orthopaedic OTs are often shared by other surgical departments, thereby limiting the opportunity to prioritize treatment for hip fractures. Other constraints were functioning imaging equipment, radiographers, implant availability and, occasionally, lack of donors for blood transfusion.

##### Motivation

Beliefs about capability and consequences—Most HCPs considered their management of older adults with hip fracture to be satisfactory and some thought that multidisciplinary approaches and best practice guideline needed closer consideration. Almost all the HCPs acknowledged the burden of hip fractures for India in the coming years and were willing to learn from the best practices. Most realize the importance of adoption of contextually appropriate models for the management of older adults with hip fractures in India to reduce mortality, morbidity and economic cost to both family and the health systems.

“I think integrated care pathways for hip fracture managements are very well established in western countries and there is a need to establish it even in our hospitals. Instead of having too many stakeholders for the beginning, you can just have orthopedic surgeons, anaesthetists and internist and these 3 or 4 people can improve the quality of care and patients can be operated earlier and the outcomes will be better”—senior orthopaedic surgeon (KII2).

Professional/social role and identity—Most the HCPs shared that orthopaedic surgeons can convince patients and their carers to accept surgery as the preferred treatment for the hip fracture. There are a group of patients with faith in traditional bone healers and aversion to surgical interventions. All the interviewed HCPs felt the need to adopt best practices but only a few were confident of their role to influence change to the existing management of older adults with hip fractures.

Optimism and intentions—The HCPs in their interviews were positive about the need to act in the right direction to improve management of hip fractures in India. Although they considered this to be an uphill task, they were optimistic as community awareness around geriatric health including hip fracture injuries is improving. Most of the HCPs thought there was scope for further improvement, but very few suggested a systems approach to adopt best practices in the management of hip fractures. There was consensus that early restoration of mobility is a necessity and participation of occupational and physiotherapy has the potential to accelerate recovery. One of the key informants believed that most of the orthopaedic surgeons prefer complicated surgeries like other joint replacements over hip fracture fixation. Even in private hospitals, there are some people who do a lot of joint replacement operations, and for them, hip fracture surgery is a low priority.

#### Healthcare providers’ recommendations to improve care pathways

Recommendations provided by the HCPs were interpreted to map six key intervention functions along with five applicable policy categories (Table 2).

**Table 2:** Healthcare providers’ recommendations to improve care pathways

	Definition	Study findings (quotes)
Intervention functions		
Education and training	Increasing knowledge or understanding Imparting skills	“I think the most important is to generate awareness and preventive care pathway, it is believed that the bone breaks post fall but it’s usually during the fall that the bone breaks, so prevent the fall”—R11 (KII) “Awareness and sensitization in terms of their roles is important and that will certainly help and improve situations if a knowledgeable and interested Physician would be a part of the initial team that is dealing with a hip fracture or any kind of a fracture patient”—R1 (KII) “I think a best exercise would be a joint training or a skill upgradation programme focused on hip fractures”—R1 (KII)
Persuasion	Using communication to induce positive or negative feelings or stimulate action	“Somebody needs to take the initiative, if you have the right willing people, you can charge people to improve practice because old people will continue to fall and they will continue to break hip”—R10 (KII)
Environmental restructuring	Changing the physical or social context	“Once such a patient is admitted, a networking system must be immediately activated with the duty of the concerned person (HCP) being coming to the patient and providing the adequate care in regards to that particular specialty. This system should be automated and referral to other department must be smooth”—R8 (KII)
		“Inter-departmental coordination should be very good, for e.g. Medicine and Endocrinology departments should coordinate very well with the department of Orthopaedics or Anaesthesia so that we all can decide to operate as early as possible”—R3 (KII)
		“There should be integrated program—my major suggestion is we should reduce the reference time, like reference from the endocrine, reference from the medicine, reference from the surgical people, that kind of integrated… so that is definitely integrated care… all the people they are together then and then it can done”—R3 (KII)
		“Probably a dedicated fracture care area, dedicated operation theatre practice, dedicated team, dedicated communication from the other segments should be... should be coordinated well”—R4 (KII)
Modelling	Providing an example for people to aspire to or imitate	“I think integrated care pathways for hip fracture managements are very well established in certain western countries and there is a need to establish it even in our hospital”—R2 (KII)
Enablement	Increasing means/reducing barriers to increase capability or opportunity	“If you increase the number of anaesthetists, naturally the theatre time will increase and once the theatre time will increase, these patients will be operated earlier. if you have two different theatres—one for neurosurgery and one for orthopaedics then naturally orthopaedic surgery will be faster and the all these patients will be operated faster”—R2 (KII) “One more very important suggestion is that we should have most of the implants here which most of the times we are not having, so that the poor people they can get those implants and we can go for surgery as they are available”—R4 (KII) “The existing infrastructure need to be upgraded along with addition of new resources as we not only have patients with fractures but also patients with spine injury, trauma and deformity competing for beds, OT time and manpower”—R13 (FGD1)
Policy categories		
Communication/marketing	Using print, electronic, telephonic or broadcast media	“We need to educate the masses and also the community healthcare workers along with PHCs and CHCs (Primary/community health care centers). Public lectures are not effective but advertising are of definite help. Live feeds or television display might be more valuable and might be picked up faster than handouts or reading materials”—R1 (KII)
Guidelines	Creating documents that recommend or mandate practice. This includes all changes to service provision	“Development of a specific standard operating procedure on what needs to be done, how these patients need to be approached, what is the minimum set of investigations that needs to be carried out and what is the minimum set of drugs that they need to go back home with”—R1 (KII)
Regulation	Establishing rules or principles of behaviour or practice	“A fracture team could be developed irrespective of the location in the hospital, if the patient presents with a hip fracture, the team needs to attend to that, in a similar manner like the cardio resuscitation team. If non-traumatic fracture reports to the hospital or if a fracture occurring out of a non-major trauma or a trivial trauma reports to the hospital, then this team could be activated. This team response may not be immediate as required in cardio pulmonary patients but say within a set of 24 hours, this team could be activated and initiates procedures according to standard operating procedure that would definitely help”—R1 (KII)
Environmental/social planning	Designing and/or controlling the physical or social environment	“Insurance should be compulsory where every Indian should be insured and the government should provide necessary subsidy. Specific policies should be made as it is a matter of financial constraint for poor people”—R3 (FGD-2)
Service provision	Delivering a service	“Ambulance service should be available at all places especially in rural areas and this should be well equipped with instruments, doctors and guidelines”—R2 (FGD-2)

The mapped intervention functions recommend the need to educate, train and persuade HCPs on multidisciplinary care for hip fracture management in their hospital settings. HCPs felt that communication between HCPs and patients or family caregivers is important in the delivery of quality healthcare as patients want doctors to not only skillfully diagnose and treat their medical condition but also communicate with them effectively. Patients should also be advised on how to protect themselves from falls in older age and making adaptive changes like, arrangement of furniture or switching on a light for visibility whilst sleeping, and support to access the bathroom from their beds (*education*, *training* and *persuasion*). The effective implementation of the standardized protocol must include prompt referral, multidisciplinary teamwork, joint training of staff, task sharing, accountability and outcome measurements. Most HCPs believed that best practice guidelines can be implemented by enabling adequate infrastructure and resources within a hospital setting. This includes increasing the number and availability of OT, implants and human resources in disciplines like anaesthesia and physiotherapy (*environmental restructuring*, *modelling* and *enablement*).

At a policy level development, interviewees unanimously agreed that there was a need to develop guidelines for hip fracture management and establish standardized care protocols in India (*guidelines* and *regulation*). Many expressed the importance of an ambulance service to transport patients from remote areas. There was consensus that district hospitals should be equipped with facilities to provide surgical care for hip fractures (*service provision*). Most HCPs felt that the surgical management of hip fractures imposes a financial burden and often leads to impoverishing expenditure for the households. There was unanimous support for community health insurance scheme like Rashtriya Swasthaya Bima Yojana, particularly for those who are poor and vulnerable, to avoid impoverishing or catastrophic expenditure from treatment for a hip fracture in an older person (*environmental* or *social planning*). Some participants suggested that there should be public awareness on osteoporosis, falls and hip fracture injury in geriatric groups using effective communication and social marketing methods, an approach to develop activities aimed at changing community perception using a variety of engagement platforms, including social media. Public information campaigns were recommended to educate on hip fracture in older persons as a life-threatening condition and the need for immediate hospitalization and early surgery. Some other key informants suggested to increase community awareness about early diagnosis and treatment of co-morbidities like hypertension, diabetes, anaemia and osteoporosis (*communications* and *marketing*) (Fig. 1).

## Discussion

The study identified significant evidence-practice gaps in the care pathway for the management of older adults with hip fractures in Delhi. Data from one hospital indicates that nearly two thirds of hip fractures were not admitted. Only a quarter of older adults with hip fractures over 80 years of age were admitted. Key informant interview and FGDs suggest a selection bias against patients with multiple co-morbidities, pressure sores and those with high risk for surgery when beds are scarce. Patients or carers refusing surgical intervention may have a low priority for admission. Such implicit biases are likely to impact key outcomes like mortality of older adults with hip fractures in the community (Table 3).

**Table 3:** Admitted vs not admitted hip fracture patients from a hospital

Patients presenting to a hospital with hip fracture (*N* = 78)		Admitted, *n* (%)	Not admitted, *n* (%)
Age (in years)	50–59	7 (26)	15 (29)
	60–69	6 (22)	12 (23)
	70–79	9 (33)	10 (20)
	80–89	4 (15)	11 (22)
	90 and above	1 (4)	3 (6)
	*t* test	−1.993 (*p* = 0.040)	
Gender	Male	14 (52)	28 (55)
	Female	13 (48)	23 (45)
	*χ* ^2^	0.066 (*p* = 0.797)	
Type of fracture	IC	11 (41)	19 (37)
	IT	15 (55)	29 (57)
	ST	1 (4)	3 (6)
	*χ* ^2^	0.224 (*p* = 0.893)	
Total		27 (35)	51 (65)

*IC* intra-capsular fracture, *IT* inter-trochanteric fracture, *ST* sub-trochanteric fracture

Our study revealed delays in admission to hospital and further delays in receiving surgery for a large proportion of older adults with hip fractures (70% did not receive surgery within the recommended period of 48 h). Late arrival to hospital and delays in receiving surgery are important evidence-practice gaps compared to the achievements in the UK of 100% admission by 24 h and surgery by 48 h for 83% [5, 10–12]. Faith in traditional bone setter could be one of the reasons in seeking care and multiple referrals [35].

Our study also found that many of the other practices, recommended in international guidelines, were not being incorporated into routine care. Findings from the KIIs and FGDs revealed that management of hip fractures is not a priority and these patients have to compete with other trauma for operating theatre availability. Lack of multidisciplinary management, overcrowding and inadequate resources were significant barriers in adopting best practices. The need for having a standardized protocol of care was considered by HCPs to be crucial for the management of older adults with hip fracture in India. There is also a need to change the perception of the healthcare providers, particularly when they are considering the management of hip fractures as satisfactory within their hospitals.

The pattern of hip fractures in this study is somewhat different to other published series. Findings revealed a higher number of men (54%) with hip fractures, compared to the preponderance of hip fractures in women elsewhere [8, 17–19, 36]. A recent hospital-based study from India also reported that a higher number of men (52%) suffered a hip fracture [37]. Our findings could be due to the relatively high number of men in Delhi where the sex ratio is 868 females to 1000 males [38].

The choice of regional anaesthesia for nearly all patients in this study is aligned to best practice recommendation from the British Society for Anesthesiologist [39, 40] and is comparable to a recent report from Beijing [23]. The types of surgical procedure and implants utilized, that were documented in this study, are similar to the practice in the UK, except for the preference of intramedullary implants for inter-trochanteric fractures in this study population. The National Institute of Care Excellence (NICE), which is a body of department of health in the UK, carries out assessments of the most appropriate treatment regimens for different disease conditions and recommends the use of dynamic hip screws for these fractures [39]. The preference for intramedullary implants might be influenced by the intensive marketing strategies of implant companies and a choice to ignore the evidence base. The choice of cemented hemi-arthroplasty for intra-capsular fracture is similar to that reported in the National Hip Fracture Database (NHFD) audits [4, 5, 10–12]. However, nearly half the patients in Delhi were mobilized by the second post-operative day, a day later than recommended [4, 39, 40].

The mean LoS in our study was 16 days, which is higher than in Sweden [41] and China [23], but lower than the 20 days LoS in the UK. The majority of people with fragility hip fracture in India are younger than in the UK [10–12], and this may explain the faster post-surgery recovery. The optimal stay in hospital following a hip fracture is still being debated with one study suggesting that LoS shorter than 10 days is associated with an increased risk of death [41], while in contrast, the Rochester co-management Model for Hip fracture showed no increases in mortality with decreased hospitalization time to 4 days [42]. Evidence from a study in Brazil suggests that delayed hospital admission for a hip fracture was associated with reduced survival at discharge and 1 year after surgery, and delay in surgery at the hospital was not found to be significant with survival [43].

Lack of osteoporosis management and falls assessment is a significant lacuna in the care pathway for hip fracture in Delhi hospitals, similar to the findings from Beijing [23]. All the patients included in the study returned home or to their original place of residence after discharge, in contrast to the UK where nearly half of the patients do not return to their usual place of residence [4, 12]. In our study, the 30-day mortality was lower than the 8% reported in the NHFD, which is part of the UK’s Falls and Fragility Fracture Audit Programme [12], but this may be an inappropriate comparison as significant loss to follow-up in our study could suggest that more patients may have died after the discharge. Information on pressure ulcer was not collected routinely in clinical practice; therefore, this adverse outcome due to delays in surgery and immobilization cannot be reported.

Our study findings acknowledge that management of older adults with hip fracture requires effective coordination across various disciplines in the hospital including rehabilitation. The co-management of hip fractures by orthopaedic and geriatric medicine has shown to be effective in achieving early surgery, mobilization and discharge from hospital with decrease in mortality [37]. Analysing the HCPs’ perspective on ways to improve practices in the management of older adults with hip fractures, the study identified six intervention functions and five policy categories to enable development of contextually appropriate areas for implementing best practices. Improving community awareness to seek urgent care for older adults with hip fracture was an identified priority as the speed of arrival will reduce time to surgery and improve outcomes [44]. Also, pre-hospital notification alone has been found to be independently associated with reduced mortality in trauma centres. There is currently no pre-hospital care in India, and the public ambulance system has only been operating for less than a decade and varies from state to state. Provision of surgical facilities at the district hospitals, ambulance service and health insurance coverage are important policy development area, particularly for the vulnerable and poor. There is a need to engage HCPs and policy makers to identify acceptable and feasible intervention strategies towards implementation of best practices for management of older adults with hip fractures in India. The benefits through a protocol-based care can generate political priority for best practice incentives, as in the developed countries, and reduce cost of care and improve health outcomes in older people with hip fractures, and these activities can also contribute to improvement in trauma care [45].

The quantitative study is based on routinely collected clinical data and, therefore, has limited information on processes, care pathways and quality indicators. For example, the time between arrival to accident and emergency department and admission to orthopaedic ward, a process indicator for best practice, is not documented routinely. Although some HCPs suggested that patients are at times admitted from the outpatient department, this information was not documented for those included in the study. Information on another best practice standards, i.e. pressure ulcer, delirium, osteoporosis and falls management, were unavailable.

### Limitation of the study

The study did not capture all hip fractures in people over 50 years reporting to these hospitals during the recruiting period. However, alongside the study, we retrospectively reviewed the only existing electronic hospital record system of a hospital to know the exact number of patients with hip fracture arrived at the emergency department during the study period (10 weeks) and discovered that a total of 78 patients arrived into this hospital out of which only 34% (27) were admitted whereas 51 were referred out. From the records, we can only know about their age group, sex, and type of fracture. Based on the information from this hospital, there was no difference in the demography of the admitted and referred patients. Key information interviews and FGD suggest a selection bias against patients with multiple co-morbidities, pressure sores and those with high risk for surgery when beds are scarce. Patients or carers refusing surgical intervention may have a low priority for admission. Such implicit biases are likely to impact key outcomes like mortality of older adults with hip fractures in the community. Lack of information on the cohort of hip fractures not admitted to hospital introduces potential biases into our findings and this is a major limitation of the study as the outcomes of hip fractures referred to other hospitals or those not seeking care are unknown. These selection biases or carer risk aversion to surgery may explain the low in-hospital mortality. Sick patients with cardiac, respiratory or renal complications are either not admitted into the hospital or may be admitted into physician or intensive care units and mortality if any is not documented as a hip fracture death.

The study compiled data at admission and then from case logs and this was a major limitation as many of the process and practices data were not routinely collected in the case logs. Future studies should include strategies to prospectively collect all relevant data on hip fracture management to enable audit and measure outcome of care pathways [1, 22]. Incorporating these minimum datasets into routine practice will enable establishing regional or national hip fracture audit and provide data for monitoring processes and outcomes. The 10-week period of recruitment from three purposely selected hospitals is a major limitation of the study. The study findings cannot be generalized to private sector hospitals with better infrastructure and human resource or to regional public hospitals with significantly limited capacity compared to the burden of injuries and poor health systems. The study does not provide information on incidence, as the denominator for hip fractures reporting to hospital was unavailable. A larger multicentre prospective cohort study would be required to understand the incidence of hip fracture in the older population in India.

We acknowledge that we could not incorporate the perspective of patients and their carers on pain, mobility and functional status in this study. This is another major limitation of the study. All patients went to their original place of residence. This information represents a lack of post-acute care facilities or rehabilitation services in India after hospital discharge. However, some patients may have access to paid nursing care and physiotherapy at their home/place of residence. Similarly, missing information on follow-ups reflects that patients either did not respond to the call or their phone numbers were wrong, even after at least three attempts of calls. This could be scenario for ‘no answer due to death’. This is again one of the limitations of the study.

## Conclusion

This study provides important information on issues beyond the need for in-hospital care pathways and evidence-based protocols for the management of older adults with hip fractures in India. The findings show huge difficulty of patients with hip fractures in having access to hospital care, from the pre-hospital services to hospital admission and surgery—which demands improvements in the entire Indian healthcare system. The development and implementation of national guidelines and standardized protocols of care for older people with hip fractures in India has the potential to improve both care and patient-related outcomes. Our mixed method study recorded vital gaps in the management of older people with hip fractures against the recommended best practices. We are able to understand key barriers and facilitators in adopting best practice evidence and potentially facilitate systematic uptake of evidence into routine practice through mapping contextually appropriate intervention and policy development areas. Wider dissemination of the findings with HCPs in a participatory manner to develop evidence-based intervention design will inform the next phase of an intervention study. Further, hip fracture audit trials can be developed to provide the evidence for data-driven policy across Delhi and India which in turn will inspire wider use of protocol-based care pathways for hip fractures and other high burden healthcare issues. Management of older adults with hip fracture requires implementation of complex interventions with co-ownership approach of the healthcare providers, policy makers, patients and others who must operationalize them beyond formal clinical setting. Health systems strengthening approach and a protocol-based care could enable achieving minimum standards of hip fracture care in India.

## Electronic supplementary material

ESM 1 (DOCX 71 kb)

ESM 2 (DOCX 99 kb)

ESM 3 (DOCX 779 kb)

ESM 4 (DOCX 778 kb)

ESM 5 (DOCX 777 kb)
