# Supplementary material for: Management of older adults with hip fractures in India: a mixed methods study of current practice, barriers and facilitators, with recommendations to improve care pathways
Source: Arch Osteoporos. 2017 Jun 2;12(1):55. doi: 10.1007/s11657-017-0344-1 (PMC5486685; doi:10.1007/s11657-017-0344-1)
Supplement: Supplementary file 1 — (DOCX 71 kb) [file 11657_2017_344_MOESM1_ESM.docx]

| \| **HOSPITAL PATIENT ID:** \| \| --- \|   **Inclusion Criteria** | **NO** | **YES** |
| --- | --- | --- | --- |
| Is the subject age 50 and above? |  |  |
| Is the subject diagnosed with hip fracture/proximal femoral fracture? |  |  |
| Is the subject willing to give written informed consent? |  |  |
| If the answer to any of these is **NO** then the patient is **NOT** **eligible** for the study | | |
| **Exclusion Criteria** | **NO** | **YES** |
| Is the subject diagnosed with a terminal stage cancer or malignancy? |  |  |
| Any other criteria for exclusion, if yes, Please specify_________________________ |  |  |
| If the answer to any of these is **YES** then the patient is **NOT eligible** for the study | | |
| **Verified and Checked by: _______________________________Date:_____________________** | | |

**SECTION-I INCLUSION AND EXCLUSION CRITERIA**

| **Surname** | **First Name** |
| --- | --- |
|  |  |
| **Date of Admission** | **Postal Address** |
|  |  |
| **Respondent Relationship** | **Preferred Telephone Number** |
|  |  |
| **Completed By** | |
| Name: Date: __\|__\|______ Signature: | |

**SECTION-II IDENTIFIABLE INFORMATION**

**SECTION-III DEMOGRAPHIC AND GENERAL INFORMATION**

| 1. Date of Birth (DD/MM/YYYY) | 2. Gender |
| --- | --- |
| __ __ __ __ /__ __/__ __  OR  Age: (Years) | Male ☐  Female ☐ |
| 3. Marital Status | 4. Are you household head? |
| Single Married  Other | Yes No  If No, then relationship with the head______________ |
| 5. Household Composition | |
| No. of people aged 15 & below ____  No. of people aged 16-59 ______  No. of people aged 60 & above _______ | |

| \| 6. Education \| \| --- \| \| Illiterate Primary  Secondary Degree/Post-graduation \| | |
| --- | --- | --- | --- |
| 7. Occupation | |
| Professional Self-employed  Laborer Unemployed  Housewife Other  Retired Please specify__________________________ | |
| 8. Carer Information | |
| Spouse/ partner  Daughter/ daughter-in-law  Son/ son-in –law | Grand children  Other unpaid help  Paid help |
| 9. Current user of Tobacco | |
| Smoking Smokeless Both  Frequency__________________/Daily/Weekly | |
| 10. Alcohol Consumption | |
| Yes No  Frequency________________/Daily/Weekly | |

| 11. Date and Time of Fall | 12. Cause of Fracture |
| --- | --- |
| Date: ___/____/_____ (DD/MM/YYYY)  Time: ___:___ (AM/PM) | Inside Home  Outside Home ☐  Missing information ☐  Fall ☐  Other occupational injury ☐ Please specify ____  Pedestrian  Car/bus accident ☐  Bicycle/Motorbike accident ☐  Missing information ☐  Road traffic injury ☐ |
| 13. Did the patient transfer from another hospital? |  |
| Yes ☐ Please specify _______________  No ☐ |  |
| 14. Where was the patient admitted from? |  |
| Home (includes the home of a family member) ☐  Nursing home with 24-hour care ☐  Transitional care ☐  Already in hospital ☐  Other ☐ Please specify __________________  Missing information ☐ |  |
| 15. Pre-facture Mobility | |
| Freely mobile without aids  Mobile outdoors with one aid  Mobile outdoors with two aids or frame  Some indoor mobility but never goes outside without help  No functional mobility (using lower limbs)  Unknown | |
