# Supplementary material for: Management of older adults with hip fractures in India: a mixed methods study of current practice, barriers and facilitators, with recommendations to improve care pathways
Source: Arch Osteoporos. 2017 Jun 2;12(1):55. doi: 10.1007/s11657-017-0344-1 (PMC5486685; doi:10.1007/s11657-017-0344-1)
Supplement: Supplementary file 2 — (DOCX 99 kb) [file 11657_2017_344_MOESM2_ESM.docx]

**SECTION-IV ADDMISSION AND ASSESSMENT**

| 1. Arrival at Emergency 0r OPD | | 2. Date and time of Transfer to Ward |
| --- | --- | --- |
| Emergency  ED admission date:  __ __ __ __/__ __/__ __  ED admission time: (HH:MM)  __ __:__ __  Yes ☐  OPD ☐time of arrival: | | Admitted to:  ICU  HDU  Emergency Ward  Orthopaedic Ward  Discharged :  Not willing for surgery  Unavailability of beds  Date: __ __ /__ __/__ ____  Time: __ __:__ __  If admitted to ICU/Emergency ward, date of transfer to orthopaedic ward __/__/____ |
| 3. Who was the attending health professional at the time of admission? | | |
| ED Doctor ☐Orthopaedic Surgeon ☐Geriatrician ☐ Any Other Please specify……………………………… | | |
| *ED- Emergency Department | | |
| 4. Bone protection medication for osteoporosis prior to admission? | | |
| Yes No | | |
| 5. Known Medical History | | |
| Previous MI Coronary Artery Disease Heart Failure  Coronary Revascularization Atrial Fibrillation Renal Failure  Dementia Stroke/Transient Ischemic Attack Diabetes Mellitus  Hypertension Depression/Anxiety  Any Other Please specify_______________________________________________________ | | |
| 6. Fracture Side Involved | 7A. Type of fracture (open/closed) | |
| Left ☐  Right ☐  Both | Open ☐  Closed ☐ | |
| 7B. Type of fracture (position) | 8. Pre-operation medical assessment | |
| Intracapsular☐  Intertrochanteric ☐  Subtrochanteric☐ | Date:  Time: | |
|  | Geriatrician ☐  Physician ☐  Specialist nurse ☐  Any Other Specialist  Please specify………………………………………..  None | |
| 9. ASA Grade | | |
| 1 ☐ 2 ☐ 3☐ 4☐ 5☐ Unknown ☐ | | |
| 10. Abbreviated Mental Test Scores (AMTS) Pre op | | |
| AMTS _/10 Not done  Patient refused | | |

| 11A. Pre-operative Investigations |
| --- |
|  |
| 11B. Investigations done post-op/or for complications |
|  |

**SECTION-V TREATMENT AND POST OPERATIVE CARE**

| 1. Pre-operative treatment | |
| --- | --- |
| Skin Traction Skeletal Traction  *Oral Analgesics………………………………….  *Intramuscular ……………………………………  *(Drug & Dosage)  Pain Control | |
| 2. Pre Anaesthesia check-up (PAC) | 3. Opinion |
| Date……/………/………….  Time after admission……. hr | Fit for surgery  Further investigation Please specify………………  …………………………………………………………….  …………………………………………………………….  Unfit for surgery Please specify the reason…  …………………………………………………………..  ………………………………………………………….  No information  Date When PAC was cleared__/__/____ |
| 4. Type of Anaesthesia | |
| Regional Anaesthesia;  Spinal ☐Epidural ☐ *General Anaesthesia* ☐  Additional Regional Nerve Block for Pain Control  Femoral nerve block ☐  Sciatic nerve block ☐  Any Other, Please specify…………………………… | |
| 5.Surgery Performed | |
| Yes ☐  Date: __ __ __ __/__ __/__ __  Time: __ __:__ __  No ☐ Please specify __________________ | |
| 6. Surgical Procedure | 7. Time from admission to Surgery |
| IM nail/ Cephalomedullary Nail ☐  DHS☐ Please specify product name ________  Hollow screw fixation/Cannulated Cancellous Screws(CCS) ☐  Hemiarthroplasty☐  THR ☐  Missing information ☐ | Less than 36 hours ☐  Between 37 and 48 hours ☐  Between 2 and 7 days☐  Longer than 7 days ☐  N/A (no surgery performed) ☐  Missing information ☐ |
| 7. Reason for delaying more than 48 hours after admission | 8. Reason for delaying more than 7 days |
| Not considered a priority surgery  Medically unfit ☐  Theatre unavailable ☐  Surgeon unavailable ☐  Anaesthetist unavailable  Other ☐  Missing information ☐ | Medically unfit ☐  Theatre unavailable ☐  Surgeon unavailable ☐  Other ☐  Missing information ☐ |
| 9. Assessed by geriatrician/physician: post-op | 10. Multidisciplinary team assessment |
| Date: __ __ __ __/__ __/__ __  Time: __ __:__ __  Yes ☐  No ☐ | Yes ☐Please specify____________________  No ☐  Missing information ☐ |
| 11. Falls prevention assessment in hospital | 12. Osteoporosis assessment and management |
| No ☐  Yes – performed on this admission ☐  Yes – awaits falls clinic assessment ☐  Yes – further intervention not appropriate ☐ | Osteoporosis assessment during hospitalization  Yes ☐  No ☐  Bone protection medication at discharge  Commenced  Continued Discontinued  Waiting |

| 13. Patient Transferred after Surgery | |
| --- | --- |
| High Dependency Unit ICU Orthopedic Ward | |
|  | |
| 14. First Day Mobilization | 15. Weight bearing after surgery |
| Yes No  Post-op Mobilization in bed  Day2 Day-------- | Partial; Day----  Full; Day----- |
| 16. Post-operative complications | 17. Mortality |
| Infection DVT  MI CVA  Pressure sores  Other, Please specify………………… | Pre-surgery Operative  Post-surgery  Cause of Death, please specify…………………..  Date_________ |
| *MI- Miyocardial Infarction DVT- Deep Vein Thrombosis CVA- Cerebro Vascular Accidents | |

**SECTION-VI DISCHARGE**

| 1. Discharge from Orthopaedic ward | |
| --- | --- |
| Date: __ __ __ __/__ __/__ __  Time: __ __:__ __  Yes ☐ Deceased  Other hospital ☐ Please specify the reason__________________________  Other speciality ☐ Please specify___________________ | |
| 2. Discharge destination from orthopaedic / hospital | |
| Own Home  carer’s home  Home with rehabilitation  Home with services  Low level residential care | High level residential care  Transitional care bed  Rehabilitation center  Other  Please specify_____________________  Missing information |
| 3. Discharge walking situation | |
| Can walk independently without aids ☐  Can walk with the use of aids (e.g. walking sticks) ☐  Cannot walk without help from another person ☐  Unable to walk  Missing information ☐ | |
