# Supplementary material for: Management of older adults with hip fractures in India: a mixed methods study of current practice, barriers and facilitators, with recommendations to improve care pathways
Source: Arch Osteoporos. 2017 Jun 2;12(1):55. doi: 10.1007/s11657-017-0344-1 (PMC5486685; doi:10.1007/s11657-017-0344-1)
Supplement: Supplementary file 3 — (DOCX 779 kb) [file 11657_2017_344_MOESM3_ESM.docx]

| **Readmission** | **Mortality** | **Care information** | **Residence** | **Bone protection** |
| --- | --- | --- | --- | --- |
| **Hip-related**  Yes   No  **Re-operation**   Yes   No  Non hip related admission:  Cause:  LOS |  Yes   No  Date of death  Recorded cause leading to death  Verbal autopsy | **Primary carer:**  Spouse/partner  Children:  Others  Paid carer  Pressure sores   Yes   No |  Home   Rehabilitation   Institution |  Yes   No  Falls prevention   Yes   No  History of fall   Yes   No |

**EQ5D**

| **Mobility** | **Self-Care** |
| --- | --- |
| I have no problems in walking about  I have some problems in walking about  I am confined to bed | I have no problems with self-care  I have some problems washing or dressing myself  I am unable to wash or dress myself |
| **Usual Activities** | **Pain / Discomfort** |
| I have no problems with performing my usual activities  I have some problems with performing my usual activities  I am unable to perform my usual activities | I have no pain or discomfort  I have moderate pain or discomfort  I have extreme pain or discomfort |
| **Anxiety / Depression** |  |
| I am not anxious or depressed  I am moderately anxious or depressed  I am extremely anxious or depressed |  |
