# Supplementary material for: Management of older adults with hip fractures in India: a mixed methods study of current practice, barriers and facilitators, with recommendations to improve care pathways
Source: Arch Osteoporos. 2017 Jun 2;12(1):55. doi: 10.1007/s11657-017-0344-1 (PMC5486685; doi:10.1007/s11657-017-0344-1)
Supplement: Supplementary file 4 — (DOCX 778 kb) [file 11657_2017_344_MOESM4_ESM.docx]

Q-1: Please tell us about the people usually admitted with hip fracture injury in your hospital

Probe: Demographic characteristics (age, sex, distance of travel); Chief compliant;

Q-2: According to you what is the usual protocol of care (pre & post op) for patients admitted to the hospital with hip fractures?

Probe: Initial reporting & referral; Care at Emergency & Orthopaedic units; physician & geriatric care; pre-anaesthetic check-up

Q-3: How do you prioritize hip fracture patients for surgery?

Probe: Factors influencing emergency Vs elective, criteria

Q-4: Please tell us more about the role of your speciality in the management of hip fractures

Probe: to understand the role each department perceive in the care pathway;

Q-5: In your opinion how can different medical specialities promote more integrative, multi-disciplinary care for hip fracture patients? (how can they work together better to manage hip fracture patients care)

Probe: Specify the departments that need to get involved; barriers for ortho-geriatric care

Q-6: Can you give us a rough estimate of the costs involved in hip fracture surgery and post-operative care within your hospital?

Probe: Hospital cost; out-of-pocket expenses; additional support available for patients in need; Elsewhere this information can be gathered

Q-7: Do you think there is a scope for improving the management of hip fracture within your hospital?

Probe: suggestion of improvement; barriers;
